# ICAnnoLncRNA: A Snakemake Pipeline for a Long Non-Coding-RNA Search and Annotation in Transcriptomic Sequences

**DOI:** 10.3390/genes14071331

**Published:** 2023-06-24

**Authors:** Artem Yu. Pronozin, Dmitry A. Afonnikov

**Affiliations:** 1 Institute of Cytology and Genetics, Siberian Branch of Russian Academy of Sciences, 630090 Novosibirsk, Russia; 2 Kurchatov Center for Genome Research, Institute of Cytology and Genetics, Siberian Branch of Russian Academy of Sciences, 630090 Novosibirsk, Russia; 3 Faculty of Natural Sciences, Novosibirsk State University, 630090 Novosibirsk, Russia

**Keywords:** transcriptome, long non-coding RNA, automatic pipeline, classification, annotation, maize

## Abstract

Long non-coding RNAs (lncRNAs) are RNA molecules longer than 200 nucleotides that do not encode proteins. Experimental studies have shown the diversity and importance of lncRNA functions in plants. To expand knowledge about lncRNAs in other species, computational pipelines that allow for standardised data-processing steps in a mode that does not require user control up until the final result were actively developed recently. These advancements enable wider functionality for lncRNA data identification and analysis. In the present work, we propose the ICAnnoLncRNA pipeline for the automatic identification, classification and annotation of plant lncRNAs in assembled transcriptomic sequences. It uses the LncFinder software for the identification of lncRNAs and allows the adjustment of recognition parameters using genomic data for which lncRNA annotation is available. The pipeline allows the prediction of lncRNA candidates, alignment of lncRNA sequences to the reference genome, filtering of erroneous/noise transcripts and probable transposable elements, lncRNA classification by genome location, comparison with sequences from external databases and analysis of lncRNA structural features and expression. We used transcriptomic sequences from 15 maize libraries assembled by Trinity and Hisat2/StringTie to demonstrate the application of the ICAnnoLncRNA pipeline.

## 1. Introduction

Long non-coding RNAs (lncRNAs) are linear or circular RNA molecules of more than 200 nucleotides in length that do not encode proteins [1]. This class of RNA is one of the most abundant in the transcriptomes of animals [2] and plants [3]. LncRNAs play essential regulatory roles such as dosage compensation effects, genomic imprinting, chromatin modification and remodelling at the chromatin level [4,5]. LncRNAs participate in cell differentiation, cell cycle regulation and regulation of alternative splicing and modulate the pathogenesis of many diseases [6,7]. In plants, lncRNAs are involved in a wide range of important biological processes. For example, COOLAIR (an antisense lncRNA) and COLDAIR (an intronic lncRNA) participate in the regulation of FLOWERING LOCUS C (FLC) that is responsible for *Arabidopsis thaliana* resistance to cold stress [8]. LncRNA AtR8 is suggested to take part in the negative regulation of translation or enzyme activity related to hypoxia tolerance in *A. thaliana* and *Brassica napus* [9]. Plant lncRNAs are involved in the response to pathogens [8] and to environmental stressors [10,11]. These and other works have demonstrated a significant role of lncRNAs in stress responses, secondary metabolism and fruit ripening in plants [12]. Therefore, the detailed characterisation of plant lncRNA functions in the cell at the molecular level is necessary. To solve these tasks, bioinformatics approaches play an important role [13]. 

Computational methods enable lncRNA prediction, filtering and functional annotation [14,15,16,17]. Identification of the potential lncRNA sequences is based on transcriptome analysis by CPC2 [18], iSeeRNA [19] and other software packages involving machine-learning methods. A large number of plant lncRNA sequences in various plant species have been identified using such methods [20]. Nevertheless, a search for lncRNAs in transcriptome data is still difficult. To date, many experiments [21,22,23] have confirmed the lower expression of lncRNAs compared with protein-coding RNAs. This finding can be explained by the fact that lncRNAs have more specific expression patterns [24] and are often restricted to certain cell types or cell lines. Furthermore, it is difficult to determine transcript sequences of low-expression genes [25]. It is worth noting that many lncRNAs have features similar to those of mRNAs (transcription by RNA polymerase II with the addition of a 5′-cap and 3′-polyadenylated tail, the presence of splicing, sequence length, frequent accumulation in the cytoplasm and an overlap with coding genes in the genome) [26,27,28]. 

Functional annotation of lncRNAs usually begins with classification, based on their genomic location relative to protein-coding genes, into intergenic, intronic, sense (transcribed from the sense strand of a protein-coding gene) or antisense (transcribed from the antisense strand of a protein-coding gene) [29]. Bioinformatics methods usually enable additional structural and functional analyses: evaluation of sequence length distribution, sequence evolutionary conservation and expression in various tissues [29]. Comparative analysis is a useful step for a better understanding of lncRNA functions [30]. Several approaches implement such an analysis in two modes: a sequence comparison within a single species [14] and a comparison between sequences from different species [30,31]. The bioinformatics approaches have been successfully applied to transcriptome analysis of several plant species: *B. napus* [15], *Zea mays* [16,31] and *Brachypodium distachyon* [14]. Large-scale transcriptome analyses have given rise to plant lncRNA databases that store the sequences and their annotations for various plant species [32,33,34,35]. It should be noted that sequences in these databases have been obtained mostly by bioinformatics tools such as CPC2 [18], CPAT [36], PLEK [37] and others. Among these databases, only the EVLncRNAs database [32], containing 4010 entries (506 for plant lncRNAs), has been built using experimentally confirmed lncRNAs from literature sources. 

To expand our knowledge about lncRNA sequences from transcriptomic experiments, several bioinformatics pipelines have been developed. They allow step-by-step data processing to be performed in a mode that does not require user control up until the final result [38,39,40]. Some of these pipelines are able to assemble a transcriptome before the lncRNA analysis [38,39,40]. Some pipelines offer an opportunity to investigate differential lncRNA expression [39]. Most of existing pipelines provide a wide range of methods for lncRNA identification and annotation. Nonetheless, they often limit the characterisation to model species only (*Homo sapiens*, *Mus musculus*, *Drosophila melanogaster* and *Danio rerio*). These limitations are partially explained by the fact that developing lncRNA recognition models use well-annotated data from a limited number of model organisms. This makes it difficult to apply most of the lncRNA analysis pipelines to fast-growing plant transcriptomic data.

In the present work, we developed the bioinformatics pipeline ICAnnoLncRNA for the identification and analysis of lncRNAs in assembled transcriptomic sequences. It uses LncFinder [41] for the identification of lncRNA sequences and allows recognition parameters to be adjusted using genomic data for which lncRNA annotation is available. Using ICAnnoLncRNA, we analysed sequences from 15 maize transcriptome libraries from different plant tissues/organs. We determined the structural features of lncRNAs and the tissue specificity of their expression and identified their homologs in a number of plant species from external lncRNA databases. We compared annotation results for two types of transcriptome assemblies, de novo and reference-based. 

## 2. Materials and Methods

### 2.1. The Pipeline for lncRNA Identification and Analysis in Plant Transcriptomes

**Figure 1:**
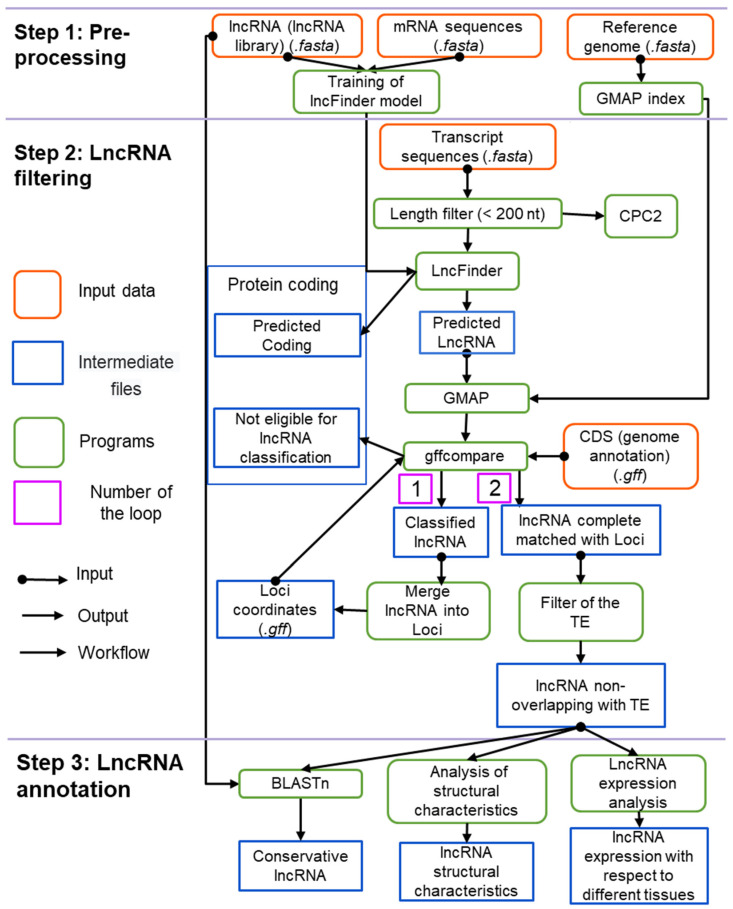
The structure of the ICAnnoLncRNA pipeline. The orange boxes show input data; the blue boxes represent results at a certain step of the pipeline; the green boxes show the software packages used at each processing step.

The computational pipeline for detecting and characterising lncRNAs in plant transcriptomes is shown in Figure 1. 

The input of the pipeline is a set of assembled transcripts provided by the user. The assembly step is not included in our pipeline and the user should obtain these sequences by de novo or reference-based assembly using additional software. Thus, our pipeline is free from the restrictions associated with the choice of a particular method of transcriptome assembling. The pipeline input also includes additional data: genomic sequences, its annotation, mRNA and lncRNA sequences annotated in the genome and description of the transcriptomic libraries (see Table S1 and Figure S1 in the Supplementary Materials).

The analysis includes several major steps: data pre-processing (panel 1, Figure 1), lncRNA identification (panel 2, Figure 1) and lncRNA annotation (panel 3, Figure 1). 

The data pre-processing step involves building an index file for genomic sequences with GMAP [42] and training the lncRNA recognition model using LncFinder v1.1.4 [41].

LncRNA identification includes: (1) prediction of lncRNA candidates in the input set of transcripts; (2) alignment of the predicted lncRNA sequences to the reference genome; (3) filtering erroneous/noise transcripts; (4) filtering possible transposable elements (TEs).

The lncRNA annotation step includes: (1) identification of lncRNA classes; (2) identification of conserved lncRNAs; (3) analysis of lncRNA expression; (4) statistical analysis. 

Details of the pipeline analysis are provided below.

#### 2.1.1. Data Pre-Processing 

This step involves building an index file for a genomic sequence with GMAP [42] and training a lncRNA recognition model using LncFinder v1.1.4 [41]. 

LncFinder enables a researcher to train a lncRNA recognition model based on known lncRNA and mRNA sequences of specific genomes with known annotation. This software uses a neural network algorithm to classify sequences and utilises three types of parameters: hexamer frequencies in nucleotide sequences, physico-chemical properties of nucleotides and features of predicted secondary structure for an RNA sequence. The sequences used for training include the full set of known mRNAs and lncRNAs for the reference genome in two separate files in FASTA format. The pipeline generates training and testing sets for the LncFinder software from this complete dataset. Taking into account that the number of known lncRNAs in annotated genomes is usually significantly smaller than the number of mRNAs (for example, 33,725 mRNAs and 2535 lncRNAs are annotated in the v.40 maize genome annotation), all known lncRNA sequences were employed for this analysis, and the number of mRNA sequences sampled by random-without-return for training and testing was assumed to be twice as large. This ratio was used in ref. [41] and ensures dataset balancing for an RNA set. 

During network training, both the mRNA and lncRNA data were randomly divided at a ratio of 80% for the training set and 20% for the test set. Using the training data, the model parameters were optimised by LncFinder. The performance of the model was evaluated on the test data using sensitivity (*SN*), precision (*PR*), specificity (*SP*) and *F1* measures [43] as follows:

*SN* = *TP*/(*TP* + *FN*), (1)

*PR* = *TP*/(*TP* + *FP*), (2)

*SP = TN*/(*TN* + *FP*), (3)

*F1* = 2 · ((*SN* × *PR*)/(*SN* + *PR*)), (4)

where *TP* (true positives) is the number of annotated lncRNAs predicted as lncRNAs, *FP* (false positives) is the number of annotated mRNAs predicted as lncRNAs, *TN* (true negatives) is the number of annotated mRNAs predicted as mRNAs and *FN* (false negatives) is the number of annotated lncRNAs predicted as mRNAs. The closer *F1* is to 1.0, the better the performance of the lncRNA prediction. All the performance measures are provided as a result of the training process at the final pipeline output.

The training and testing procedures described above were performed in five replicates. The sequences for each replicate were chosen independently. The pipeline outputs performance estimates for lncRNA recognition for each replicate in a separate file in a CSV format. The pipeline saves the parameters of the replicate having maximal *F1* in a separate file and uses them for further analysis. 

The LncFinder package offers the opportunity to use secondary structure parameters estimated by the ViennaRNA software [44] for lncRNA recognition. Our pipeline supports this option. In this case, the calculation time is usually increased by ~95%. At the same time, according to our preliminary estimates (data not shown), the performance of the lncRNA recognition improves by ~1% only. Therefore, in this work, we did not use secondary structure parameters to predict lncRNA candidates.

#### 2.1.2. LncRNA Identification and Filtering

LncRNA identification includes four steps: (1) prediction of lncRNA candidates in the input set of transcripts; (2) alignment of the predicted lncRNA sequences to the reference genome; (3) filtering erroneous/noise transcripts; (4) filtering possible transposable elements (TEs).

The lncRNA recognition was performed by LncFinder [41] with the parameters determined at the previous step (see above). Transcripts were classified into two sets—coding and non-coding sequences—and the classification data stored in an output text file in TSV format. The sequences identified by LncFinder as non-coding were lncRNA candidates. For each transcript, the ORF length, the Fickett index, ORF coverage and the isoelectric point were estimated by CPC2 [18] and stored in a separate file as additional information. These parameters, however, did not affect the lncRNA prediction results. 

Alignment of the lncRNA candidates to the reference genome was performed using the GMAP software v2020.10.14 [42] with the following parameters: —min-intronlength = 1 —intronlength = 414,579—cross-species—format = gff3_gene—split-large-introns—npaths = 1.

In the pipeline, we applied filtering to short sequence fragments resulting from de novo assembly errors or transcriptional noise. For this purpose, we identified genomic lncRNA loci as a continuous region of a chromosome to which at least one lncRNA transcript is aligned. The GFF file with lncRNA loci coordinates is used by the gffcompare tool v0.11.2 [45] to select transcripts that matched the loci regions (class ‘=’ transcripts, see below); transcripts without a match to lncRNA loci were removed from further analysis.

A second filter is aimed at removing transcripts from transposable elements loci. The user should provide a GFF file with TE coordinates in the reference genome as input to the ICAnnoLncRNA pipeline. All candidate lncRNA transcripts that overlapped by at least one nucleotide with the TE region were removed from further analysis. In our work, we used the EDTA package [46] to obtain TE libraries for the maize AGPv4 genome and to identify TEs in its sequence.

Candidate lncRNAs sequences that passed these filters were analysed further by the ICAnnoLncRNA pipeline.

#### 2.1.3. Identification of lncRNA Classes 

To classify the candidate lncRNA sequences by their location in the genome relative to protein-coding genes, ICAnnoLncRNA uses the program gffcompare v0.11.2 [45]. Each transcript that passes through filters at the previous steps is classified by this program into 1 of 14 classes represented by single-character notation. LncRNA candidates that completely matched known mRNA sequences (class ‘=’) due to possible prediction errors were excluded from further analysis. Some lncRNA candidates aligned with exons in the ‘+’ direction (classes ‘c’, ‘k’, ‘j’, ‘m’, ‘n’ and ‘o’). There is experimental evidence in humans that several lncRNAs are encoded by exonic fragments in the ‘+’ direction. However, these cases are infrequent, and no such examples are currently known in plants. In addition, it has been suggested that transcripts of the ‘c’, ‘k’, ‘j’, ‘m’, ‘n’ or ‘o’ class may represent new isoforms of known genes rather than lncRNAs [47]. For the above reasons, the sequences of these classes were excluded from further analysis. Some lncRNA candidates belong to classes ‘e’, ‘s’, ‘p’ and ‘y’ (including joined intron and exon fragments). We believe that these sequences are not lncRNAs and mainly represent sequencing/assembly artefacts. They were excluded from further analysis by our pipeline. 

Some sequences of the ‘=’ class aligned to known lncRNA genes annotated in the reference genome. Transcripts not included in the above classes were regarded as novel lncRNAs. The pipeline provides analysis of known as well as novel lncRNAs. However, in the current work, we focus our analysis on the detection of novel sequences not presented in the current genome annotation. 

**Figure 2:**
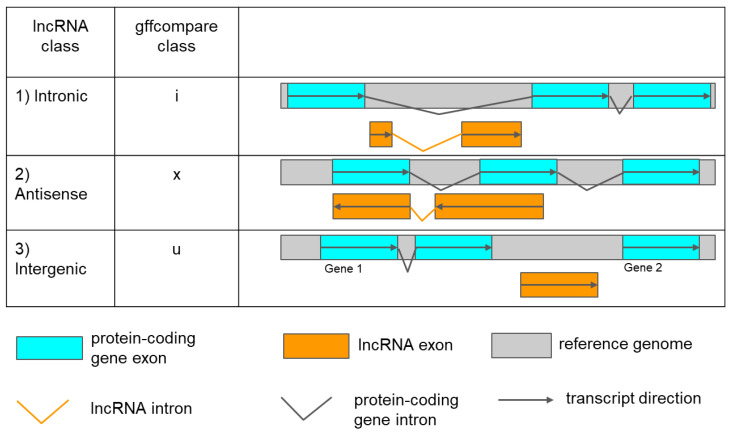
Identification of three types of lncRNAs by their positioning relative to protein-coding genes according to gffcompare classification. The orange bars indicate lncRNAs exons, and the grey represents one reference genome. Cyan bars indicate mRNA exons. Broken orange and black lines show lncRNAs and protein-coding gene introns, respectively. An arrow shows the direction of transcripts.

Novel lncRNAs were categorised into three types [14,29,48] (Figure 2):

- intronic: a lncRNA that is fully contained within a reference intron (gffcompare class ‘i’).
- antisense: an exonic overlap on the reverse strand of a protein-coding gene (gffcompare class ’x’).
- intergenic: a lncRNA that is located between two protein-coding genes (gffcompare class ‘u’).

In accordance with the classification of lncRNAs described above, we categorised their loci into two types: intergenic lncRNA loci (class ‘u’) and loci overlapping with known protein-coding genes (classes ‘i’ and ‘x’).

#### 2.1.4. Identification of Conserved lncRNAs

Similarly to ref. [31], the ICAnnoLncRNA pipeline classifies novel lncRNAs as conserved and non-conserved by the presence/absence of their homologs in other species in the external databases. The lncRNA sequence from the reference species was considered conserved if a lncRNA sequence with identity greater or equal to 50% was found in other species in the dataset of known lncRNAs (see Section 2.4 below). The search was performed using BLASTn v2.9.0+ [49] with the parameters: -outfmt 6 -evalue 1e-50 -max_target_seqs 1 -perc_identity 50. Otherwise, the lncRNA was regarded as non-conserved. 

The pipeline also allows the clustering of novel lncRNA sequences and sequences from the dataset of known lncRNAs by UCLUST v. 11 [50]. This allows the identification of groups of homologous lncRNAs from different species. 

#### 2.1.5. Analysis of lncRNA Expression 

The pipeline allows the specificity of lncRNA expression in various plant tissues to be determined using the data provided by user at the pipeline input (Table S1, Supplementary Materials). Our pipeline can compare three sets of RNA sequences: two sets of lncRNAs (conserved and non-conserved) and transcripts predicted as protein-coding. For each of the three RNA types, the number of sequences expressed in at least one tissue is estimated (expression value is greater than user-defined threshold). The number of transcripts of each class expressed in a given tissue is normalised to the number of transcripts of a given RNA type and to the number of experiments relevant to that tissue. The proposed normalisation takes into account the unequal number of genes represented in the three types of RNAs and the unequal number of experiments performed on different tissue types. This value (the number of expressed transcripts per library per tissue) shows the specificity of gene expression of the selected RNA molecules (conserved lncRNAs, non-conserved lncRNAs and mRNAs) for each tissue analysed. However, this value should not be confused with the overall expression level of the specific molecule type in the tissue. 

#### 2.1.6. Statistical Analysis

Our pipeline outputs several important statistics for novel lncRNAs: density in each chromosome, number of different classes of genome location, distribution of exon/intron characteristics, and specificity of expression in different tissues. During the analysis of the lncRNA exon/intron structure, we excluded those lncRNA transcripts with a small intron length (<60 bp) which could be obtained for de novo assembled transcripts but would be highly unlikely, according to experiments [51]. These transcripts, however, were not excluded from the analysis of expression and homology. 

### 2.2. The Platform

The pipeline was designed using the workflow management system Snakemake v6.0.0 [52]. Snakemake is a tool for devising reproducible and scalable data analyses. Workflows are described in a human-readable, Python-based language. They can be seamlessly scaled to server, cluster, grid and cloud environments without the need to modify the workflow definition. Snakemake is compatible with the Conda management system (https://docs.conda.io/en/latest/, accessed on 1 October 2022), which enables an investigator to easily install any new programs needed for the pipeline. The direct acyclic graph (DAG) for the ICAnnoLncRNA pipeline is provided in Figure S2 (Supplementary Materials) and shows its implementation in detail.

### 2.3. Transcriptomic Datasets and Sequence Assembly

We analysed 15 maize transcriptome libraries (Table S2, Supplementary Materials) from different plant tissues/organs. Two approaches were used for sequence assembly. The first one was the de novo method and included extraction of reads from SRA files by SRA Toolkit [53], data pre-processing by fastp [54], sequence assembly by Trinity v2.6.6 [55] and transcript quantification in TPM (transcripts per million) by Kallisto [56] (the analysis is described in detail in [57]). The second approach was reference-based. Reads were filtered and mapped to the reference maize genome AGPv4 [58] retrieved from the Ensembl Plants database [59] by Hisat2 v. 2.1 [60] and assembled by StringTie v. 1.3 [61]. We used the implementation of the second approach from the LncPipe pipeline [39]. We used Ensembl Plants v.40 annotation files for the reference genome. 

The analysis of tissue expression specificity for the transcripts was performed in this work for transcripts identified in the Trinity assembly. The transcript was considered as expressed if its TPM was greater than 1.

### 2.4. A Library of Known lncRNA Sequences 

Sequences of known lncRNAs from PNRD [33], CANTATAdb v2.0 [62], GREENC v1.12 [35], PLncDB v2.0 [34] and EVLncRNAs v2.0 [32] databases were used for comparison with the lncRNAs obtained in the current work for maize transcriptomes. The sets of sequences from these databases were combined; identical sequences were removed to obtain a non-redundant sequence dataset. This procedure yielded 256,091 lncRNA sequences from 16 plant species; 39,456 of these belong to maize. Detailed statistics on the number of sequences of each species in the non-redundant dataset are presented in Table S3 (Supplementary Materials).

## 3. Results

### 3.1. LncRNA Identification

In this study, a set of 2535 sequences of known lncRNAs and 33,725 maize mRNAs from the Ensembl Plants v.40 annotation [59] were used to train the lncRNA prediction model (see Section 2.1.1). The best performance of the LncFinder prediction in five replicates was achieved at *F1* = 0.91. 

The results of step-by-step processing of two transcriptome assemblies for 15 maize RNA-seq libraries is shown in Table 1. The table demonstrates that Trinity assembly yields almost six time more transcripts than the Hisat2/StringTie assembly. However, the number of transcripts identified by our pipeline as lncRNA candidates is almost 20-fold larger for the Trinity assembly. The same ratio holds true for lncRNA loci.

Our analysis of lncRNA transcript alignments identified 52,526 loci for the Trinity assembly and 3583 loci for the Hisat2/StringTie assembly (Table 1). The lncRNA loci in total comprise 19,525,698 bp (1% of the genome) for the Trinity assembly and 1,799,826 bp (0.08% of the genome) for the Hisat2/StringTie assembly. Our results show that on average, the number of transcripts expressed from one lncRNA loci is similar for both Trinity and Hisat2/StringTie assemblies: 1.16 and 1.03, respectively. 

**Figure 3:**
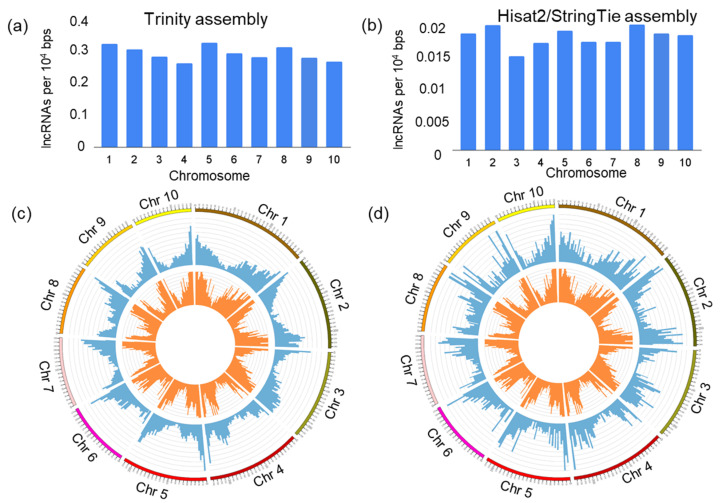
The distribution of identified novel lncRNA loci across maize chromosomes. (**a**,**b**) Density of lncRNAs (*Y*-axis) in each chromosome (*X*-axis). (**c**,**d**) The distribution of lncRNA (blue colour) and protein-coding genes (orange colour) loci by chromosome length as a circular chart. The distributions for Trinity and Hisat2/StringTie assemblies are shown in (**a**,**c**) and (**b**,**d**), respectively.

The distribution of lncRNA loci density within the maize genome is shown in Figure 3a,b. The highest density of lncRNAs is observed for chromosomes 1, 5 and 8 for the Trinity assembly and chromosomes 1, 2 and 8 for the Hisat2/StringTie assembly. The smallest density is observed for chromosomes 3 and 4 for both assemblies. The results show that the distribution of lncRNA loci along the chromosomes is uneven. The frequency of occurrence of these loci is higher towards the chromosomal termini and smaller at the centromeric regions (Figure 3c,d). This tendency is similar for both assemblies.

### 3.2. LncRNA Classification

LncRNA candidate transcripts were classified by ICAnnoLncRNA pipeline into three classes: antisense, intron and intergenic, as described above. The number of transcripts belonging to these classes are shown in Table 2. The number of transcripts corresponding to the known lncRNAs is provided in the last row. 

The ratio of antisense, intronic and intergenic lncRNAs is 56:27:15 for the Trinity assembly and 42:4:52 for the Hisat2/StringTie assembly. Clearly, the second method of assembly yields a much smaller fraction of predicted intronic lncRNAs and a larger fraction of intergenic lncRNAs. Interestingly, the number of expressed transcripts for known lncRNAs is similar for both methods of assembly (306 and 297). 

### 3.3. Analysis of lncRNAs’ Structural Characteristics 

**Figure 4:**
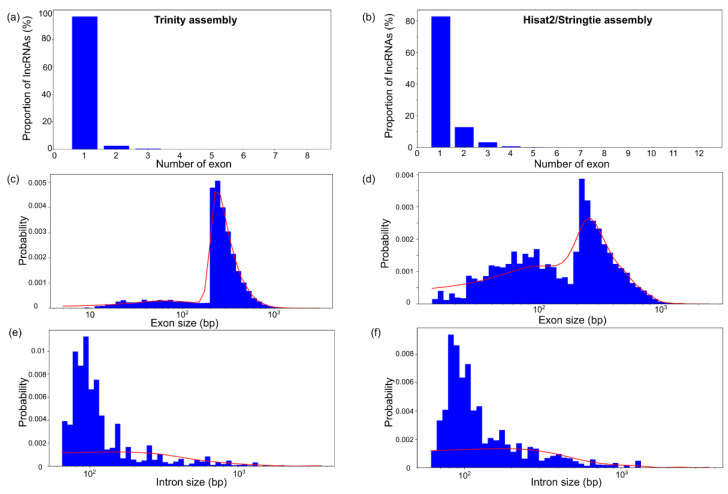
The distribution of structural characteristics of the maize lncRNA transcripts. (**a**,**b**) The distribution of the number of exons per lncRNA. (**c**,**d**) The distribution of exon length. (**e**,**f**) The distribution of intron length. The distributions for Trinity and Hisat2/StringTie assemblies are shown in (**a**,**c**,**e**) and (**b**,**d**,**f**), respectively.

Transcript alignment to the reference genome enables the identification of the exon–intron structure of the novel loci encoding lncRNAs. The distribution of these loci by the number of exons, exon length and intron length is displayed in Figure 4. 

The analysis revealed that more than 80% of lncRNAs contain a single exon for both assemblies (Figure 4a,b). However, Hisat2/StringTie assembly transcripts demonstrated a larger fraction of loci with two and three exons (up to 10% for two-exon transcripts). The distribution of lncRNAs by exon length has two characteristic regions. A small proportion of exons are up to 200 bp long, but the vast majority are between 300 and 1000 bp long. The fraction of lncRNA transcripts from the second region of the exon size distribution is larger for the Trinity assembly than for the Hisat2/StringTie assembly. The distribution of intron lengths for the lncRNAs is similar for both types of assembly; the peak is around 100 bp and there exist some long introns (~1000 bp).

Statistical characteristics of the structural organisation of known maize lncRNA loci and novel lncRNAs are presented in Table 3. These characteristics are represented by the mean and median of the exon length distribution and the mean and median of the intron length distribution. The mean transcript length is an exception, and it is almost one and a half times as large for the Hisat2/StringTie assembly. 

On the other hand, there are lncRNA characteristics that differ between the Trinity and Hisat2/StringTie transcriptome assemblies. These are mostly related to extremal (maximal and minimal) lengths of the exons and introns (except the maximal intron length) (Table 3). The minimal values for exon and intron lengths for the Trinity assembly were 3 to 61 times smaller, respectively, than for the Hisat2/StringTie assembly. 

Table 3 allows the comparison of statistical characteristics for known and novel lncRNAs. Interestingly, for exon length distribution, the mean/median values are very similar. The minimal exon lengths of the known lncRNAs and the lncRNAs assembled by Trinity are also very similar. The exception is the known lncRNAs’ maximal exon length, which is 2.5 and 3.6 times larger than for the Trinity and Hisat2/StringTie assemblies, respectively. These remarkable differences are observed for intron-length statistics. All the parameters except the minimal length are several times larger for known lncRNAs. 

**Figure 5:**
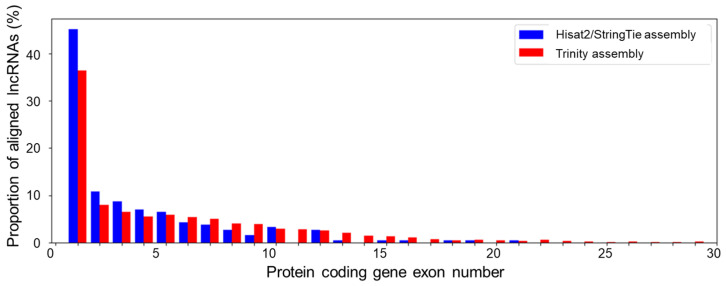
The distribution of antisense lncRNA alignments by the matching protein-coding-gene exon number. The correspondence of bar colour to the type of assembly is shown in the upper left corner of the diagram.

For novel antisense lncRNAs, we examined the distribution of the number of lncRNAs aligned to different exons of protein-coding genes (Figure 5). The proportion decreases sharply with the exon number. More than 35% of lncRNA transcripts aligned to the first exon (for both assemblies). Up to 10% of transcripts aligned to the second exon. The distribution of the fraction of aligned transcripts for the third and other exons decreases slowly. 

### 3.4. Analysis of lncRNA Conservation

We compared sequences of the novel lncRNAs with sequences of known lncRNAs from external databases (see Materials and Methods, Section 2.4). The high variability of lncRNA sequences is supported by the results of clustering by the UCLUST program of the complete set of novel lncRNAs with sequences from external databases (similarity threshold of 50%; Table S4, Supplementary Materials). This yields more than 240,000 clusters for both the Trinity and Hisat2/StringTie assemblies. Moreover, the median value of the number of sequences is one to two for clusters consisting of either external sequences or lncRNAs obtained in this work. For clusters comprising both type of sequences, the median size is four.

Our results demonstrate high specificity of the novel lncRNA sequences to maize (Table 4). 

A large fraction of the lncRNAs we obtained is specific to our transcriptomic datasets (86% have no homologs with sequences from external databases). The largest fraction of candidate lncRNAs have homologs to maize sequences from external databases (14% for Trinity and 30% for Hisat2/StringTie assembly). The proportion of sequences homologous to lncRNAs from organisms other than maize is about 1% (Table 4). This implies that the majority (~99%) of candidate lncRNA sequences are specific to maize. The fraction of lncRNAs in our dataset with homologs in other species decreases sharply with the increase of the evolutionary distance from maize (Table S5, Supplementary Materials): ~6% for *S. bicolor*, ~2% for *O. sativa*, ~0.1% for *B. distachion*. 

**Figure 6:**
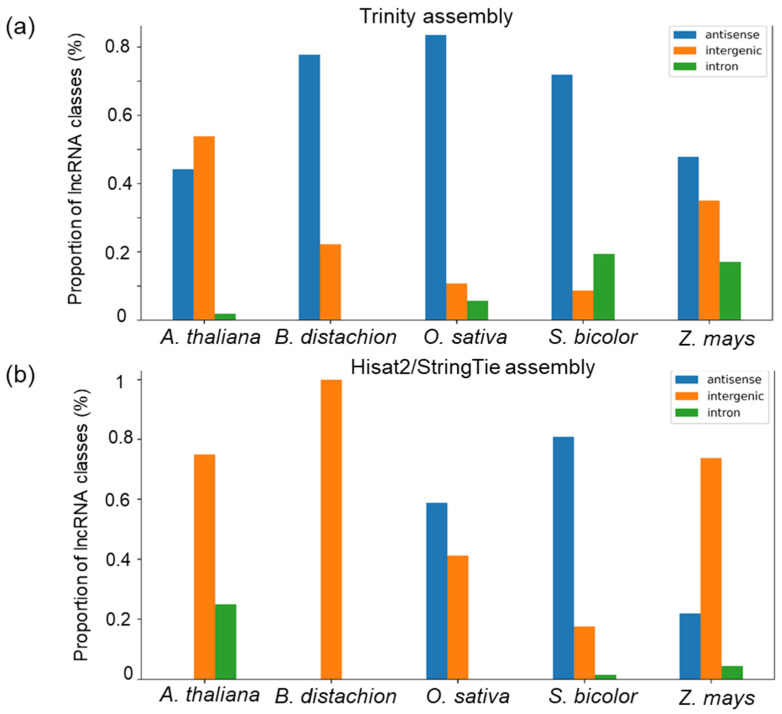
The distribution of novel maize lncRNAs identified in the Trinity (**a**) and Hisat2/StringTie (**b**) assemblies among the three classes, depending on the presence of similar sequences in various species from external databases. The *X*-axis represents species. The *Y*-axis represents the class proportion (%) in a set of the novel lncRNAs with similarity to lncRNAs from these species in the external databases. The correspondence of bar colour to the lncRNA class is shown in the upper left corner of the diagram.

Figure 6 illustrates the distributions of different classes of maize lncRNAs depending on their similarity to lncRNAs from other organisms. Interestingly, two assemblies differ remarkable by this distribution. The Trinity assembly demonstrates that the largest fraction of the antisense lncRNAs is homologous to sequences from the monocot species. The exception is *A. thaliana*, with a higher proportion of homologous intergenic lncRNAs. The fraction of intronic lncRNAs with homologs in other species is small and decreases with the evolutionary distances from other species. 

LncRNAs obtained by the Hisat2/StringTie assembly demonstrate the highest fraction of intergenic sequences homologous to *A. thaliana*, *B. distachion* and *Z. mays* sequences from external databases. The fraction of antisense lncRNAs is higher for homologs from *O. sativa* and *S. bicolor*. The fraction of intronic lncRNAs with homology to other species is small. 

### 3.5. LncRNA Expression Analysis in Different Maize Tissues

**Figure 7:**
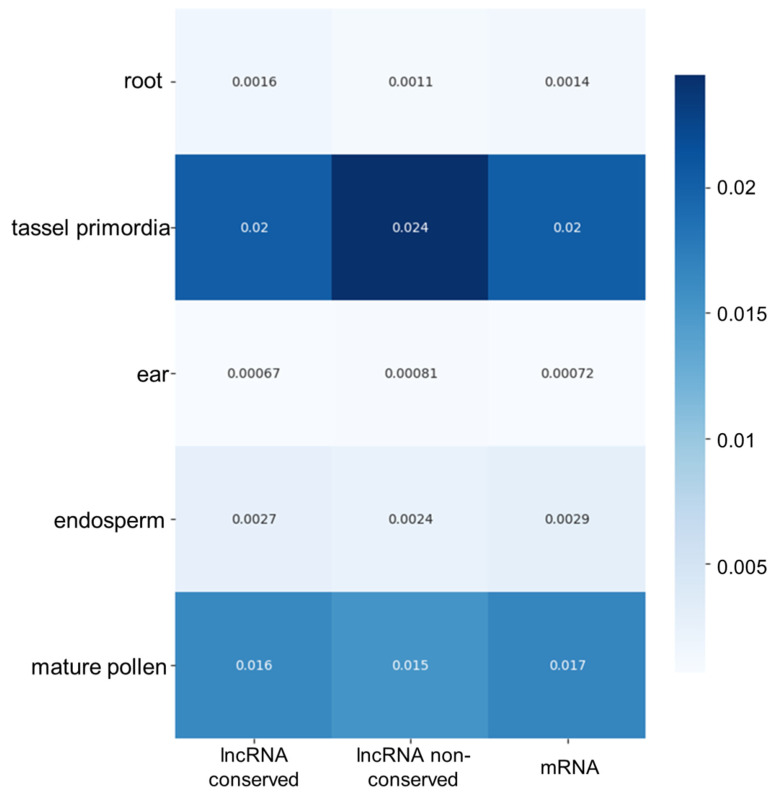
Specificity of lncRNA expression in different maize tissues, presented as a heat map. The *X*-axis indicates data for two classes of lncRNAs (conserved and non-conserved) and mRNAs. The correspondence of the cell colour to specificity values is shown by the scale on the right-hand side.

We evaluated the expression specificity of lncRNAs among different tissues and organs of the plant. We performed this analysis both for conserved and non-conserved lncRNAs and, for comparison, for mRNAs identified in the transcriptomes. The results are shown in Figure 7. According to our data, the lncRNA expression specificity is higher for tassel primordia and mature pollen (both for conserved and non-conserved lncRNAs). This specificity is at least 10 times higher than for other tissues. Interestingly, we observed the same pattern of specificity between tissues for mRNAs. 

## 4. Discussion

Experimental studies have shown the diversity and importance of lncRNA functions in plants: participation in the modulation of gene expression, homeostasis of plant physiological parameters, formation and development of various tissues and organs and responses to biotic and abiotic stressors. To date, over half a million lncRNA sequences have been identified in various plant species [34]. Due to the rapid growth of information on whole-genome and transcriptome sequences from different varieties and lines of important crops, the number of identified lncRNAs continues to grow. Nonetheless, structural and functional features are known only for a small number of lncRNAs and are experimentally confirmed only in a few cases. Therefore, there is a need to create efficient bioinformatics pipelines for a large-scale search and for the structural and functional annotation of lncRNAs in agricultural plants. 

The existing lncRNA identification and annotation pipelines lncEvo [38], LncPipe [39] and CALINCA [40] have been developed for human and animal transcriptome analysis. The workflow of all pipelines for non-coding RNA sequences involves their identification and functional analysis. The CPC2 program [18] is used to identify lncRNAs in most of the above-mentioned pipelines. It divides a set of studied transcripts into coding and non-coding sequences on the basis of four features related to an ORF. Unfortunately, when recognising non-coding sequences, the program manifests a rather high error rate [16,36,63]. In our work, we chose the LncFinder program, which has higher accuracy compared with CPC2 [18]. In addition, the advantage of LncFinder is that it is based on machine-learning algorithms in which the parameters for a specific reference genome can be selected individually. Our pipeline implements this capability using reference genome data on annotated lncRNAs. This approach offers greater accuracy in the identification of lncRNAs in various plant species, whose genome structure is diverse [64]. Note that unlike deep-learning methods [63], the LncFinder algorithm does not require a very large dataset for training. This feature is useful when one is creating a recognition method based on annotation data from a reference genome containing a limited number of known lncRNAs (even for the well-studied maize genome, this number is ~2000). During parameter selection, LncFinder evaluates the accuracy of the lncRNA recognition; for the maize genome that we analysed, this was high (*F1* = 0.91).

The next step in most pipelines is to filter candidate sequences, because lncRNA recognition inevitably contains errors. LncPipe applies an additional filter to transcript length (greater than 200 nt) and to the number of exons (greater than 2). CALINCA and lncEvo have similar filtering criteria: sequence length (>200 nt), expression level (FPKM > 1, TPM > 1, respectively) and ORF length (should not exceed 50 amino acid residues (codons) (CALINCA) or 100 codons (lncEvo)). In our case, we use a filter that excludes sequences expressed from TE regions and isoforms of small sizes (possible noise). 

The next filtering step involves aligning the sequences to a reference genome and comparing that alignment with the annotation of known genes (protein-coding and non-coding). We accomplish this task by means of the gffcompare program, which is actively used in lncRNA identification [48,65,66,67,68]. It allows the rejection of transcripts that completely match genes of known proteins (likely a consequence of inaccurate prediction of lncRNA candidates). The next large group of sequences identified by this method are the fragments that overlap in the positive direction with exons of protein-coding genes. Some researchers consider such sequences as lncRNA candidates [15,38]. Nevertheless, a number of authors believe that such fragments can correspond to new isoforms of known genes [48,68]. In our pipeline, we chose a more robust approach to lncRNA identification and excluded transcripts of this type from the analysis. Thus, we offer the user the most reliable set of transcript sequences suspected of encoding lncRNAs. 

As in most other research articles in this field, we analyse three types of detected lncRNAs: intergenic, intronic and antisense. In the bioinformatics pipelines mentioned above, the cuffcompare software [69], which is an analogue of gffcompare, is utilised to classify lncRNAs.

After lncRNA identification, most pipelines offer the user an additional transcript analysis. The lncEvo pipeline can perform a conservation analysis between two organisms of interest. CALINCA can analyse differential expression and lncRNA expression in different tissues and conservation in relation to other organisms. LncPipe offers differential expression analysis. Our pipeline implements most of the above features and allows for an analysis of lncRNA structural features (exon–intron structure and features of antisense alignment on protein-coding sequences). At the same time, the user is permitted to input his/her own data on both expression levels and external lncRNA sequences. 

Our pipeline uses as input a set of transcriptomic sequences provided by the user. Thus, it is possible to choose any method of assembly suitable for the user’s task. Some users may be interested in reference-based transcriptome assembly. Others may prefer de novo assembly, even when the reference genomic sequence is known and well annotated. For example, generating de novo transcriptome assemblies for model plants such as *A. thaliana*, rice and maize could be useful for discovering new transcript isoforms of existing annotated genes and alternative splicing events as well as novel transcribed genes from a plant variety or in response to a specific treatment [70].

Taking into account these possibilities, in this study, we performed an analysis of transcripts from 15 maize RNA-seq libraries assembled by two methods (de novo and reference-based) to demonstrate the suitability of our pipeline for both types of data. The overall data analysis by the ICAnnoLncRNA pipeline took 2 h for the Trinity assembly and 1 h 30 min for the Hisat2/StringTie [16,31] assembly using a cluster node with 1 TB of RAM and 32 cores of the AMD EPYC 7452 processor. As result, we obtained a number of important characteristics of maize lncRNAs describing their primary structure, location in the genome, similarity to lncRNAs from other species and expression tissue specificity. 

A comparison of lncRNA characteristics for assemblies obtained by different methods demonstrates both their similarities and differences. Both assemblies demonstrated common features in the distribution of lncRNA loci along the chromosomes: the frequencies of occurrence are higher for the termini and lower for centromeric regions of the chromosomes. This looks reasonable due to high proportion of antisense and intronic lncRNAs we identified (50% and higher for both assemblies). These lncRNAs are co-located with protein-coding genes, closer to the chromosome termini [58]. Our results differ from previously published works [16,31] in which the lncRNA distributions along the chromosomes are almost uniform. These differences may arise because we performed additional filtering of those transcripts associated with transposable elements located with high frequency in the centromeric regions of the chromosomes [58]. It is also interesting that both of our assemblies contained approximately the same number of transcripts for known maize lncRNAs.

Another parameter for which the values were very similar for both assemblies was the number of transcripts expressed per lncRNA locus (~1). This value is close to estimates from other studies on lncRNA search and analysis in maize [16], *A. thaliana* [17] (~1.6 transcripts per locus) and tomato [71] (~1.6 transcripts per locus). It should be also noted that lncRNAs having a single exon dominate both types of assemblies. This characteristic feature was also found earlier in [16].

It is also interesting that the mean/median values for the distributions of exon and intron lengths are also similar for both assemblies (~250 bp for exons and ~100 for introns). The estimates of the mean lncRNA transcript length are also close (380–500 bp). They approximate to the estimate of 463 bp obtained in ref. [16]. A low proportion (more than 6-fold) of intronic and a high proportion of intergenic lncRNAs detected by the Hisat2/StringTie assembler is also associated with this feature (Table 2). 

The estimated proportion of intergenic lncRNAs for the transcriptomes we studied appears to be lower than in other studies. For instance, in the study by Li et al. [16], 93% of the 1704 corresponding transcripts were classified as intergenic lncRNAs and 7% as antisense. Those authors did not examine intronic lncRNAs. Han et al. [31] analysed 400,000 transcripts and identified 18,000 lncRNAs. Those authors classified 84% of the sequences as intergenic, 7% as antisense and the remaining 3% as intronic. Examination of lncRNAs in *B. distachyon* and *B. napus* [14,15] shows the predominance of the intergenic lncRNA class at 81% and 88%, respectively. Similar to our results, analysis of lncRNA transcripts in *Populus trichocarpa* and *A. thaliana* indicated a predominance of the antisense class [72,73], while the intronic class was the least abundant.

We searched for sequences with similarity to our lncRNAs among sequences from external databases. We found only 1% of our lncRNAs with similarity with lncRNAs in other plant species. Not surprisingly, the largest numbers of homologues were found in the monocotyledonous plants, *S. bicolor* and *O. sativa*. This is similar to results obtained by Han et al. [31], who demonstrated only 2.5 and 0.1% of maize sequences as homologous to known lncRNAs from sorghum and rice, respectively. Our results are in agreement with conclusions from previous studies: lncRNA sequences are highly species-specific, and their similarity with lncRNAs from other species drops sharply with increasing evolutionary distance [10,30]. 

Homologous sequences in other species were more frequently found for antisense lncRNAs for the Trinity and intronic lncRNAs for the Hisat2/StringTie assembly. The explanation could be simply the higher occurrence of these classes of lncRNAs in the respective assemblies. These results are in agreement with the work of Deng et al. [10]. Those authors compared the lncRNAs identified by computer methods from *A. thaliana* and *O. sativa* with those from 10 plant species and noticed that the closer two species are on a species phylogenetic tree, the more similar lncRNA sequences they have. Overall, our analysis indicates that lncRNA sequences are highly species-specific. In comparison with various plant species, the highest proportion of our transcripts shares similarities with already known lncRNAs from maize. Our results also demonstrate the high variability of the lncRNA sequences found (Table S5, Supplementary Materials).

Our examination of the expression of novel lncRNAs points to the high expression of conserved and non-conserved lncRNAs in the mature pollen and tassel primordia of maize. Interestingly, the specificity of mRNA expression in our datasets is the same. Note that our results could not be compared directly with that of Han et al. [31], who estimated the fraction of transcripts of different types within the same tissues. In our work, we used additional normalisation based on the number of expressed transcripts in all tissues, which we suggest is more relevant for the expression specificity determination. 

## 5. Conclusions

We propose a bioinformatics pipeline for automated lncRNA prediction, classification and annotation based on an analysis of transcripts from RNA-seq libraries. The pipeline uses the LncFinder method for the recognition of lncRNA candidates while allowing for the selection of recognition parameters for a given genome, thereby significantly improving the accuracy of lncRNA recognition. Due to the introduced filtering system (alignment to a reference genome, removing transposable elements, filtering short isoforms and classification), the pipeline outputs a reliable set of transcript sequences that are presumably lncRNAs. The pipeline also enables the subsequent annotation of novel lncRNAs (a similarity search among sequences from external databases, expression analysis, and assessment of the structural features).

The pipeline was applied to the analysis of 15 maize transcriptome libraries assembled by de novo and reference-based methods. We demonstrated that the results of the identification and structural annotation of lncRNAs depends on the assembly method. Transcripts were identified that share similarities with lncRNAs from other plant species. Evaluation of evolutionary conservation revealed that lncRNA sequences are predominantly species-specific; in comparison with various plant species, the highest proportion of the transcripts had similarities with already known lncRNAs of maize. Analysis of the expression of newly detected lncRNAs showed specificity of expression of conserved and non-conserved lncRNAs in the tassel primordia and mature pollen. 

## Figures and Tables

**Table 1:** Identification of lncRNA candidates by the ICAnnoLncRNA pipeline in 15 maize transcriptome libraries assembled by Trinity and Hisat2/StringTie. The percentage of transcripts relative to the previous analysis step is shown in parentheses.

Pipeline Step	Trinity Assembly	Hisat2/StringTie Assembly
Input	656,289	91,721
Length filtering	656,289 (100%)	91,721 (100%)
LncFinder	450,504 (69%)	21,186 (23%)
Genome alignment	441,733 (67%)	18,428 (20%)
Filtering errors/noise	101,207 (15%)	9837 (10%)
Filtering possible TEs	40,203 (6%)	6128 (6%)
lncRNA candidate transcripts	61,004 (9%)	3706 (4%)
lncRNA candidate loci	52,526 (8%)	3583 (4%)

**Table 2:** Number of lncRNAs in the three classes of localisation relative to protein-coding genes.

Classes	Gffcompare Class	Trinity Assembly	Hisat2/StringTie Assembly
antisense	x	34,611 (56%)	1590 (42%)
intron	i	17,043 (27%)	180 (4%)
intergenic	u	9350 (15%)	1936 (52%)
Known lncRNA loci	=	306	297

**Table 3:** Comparison of the structural features between known maize lncRNAs and novel lncRNAs.

Structural Characteristic	Known lncRNAs	Novel lncRNA	
		Trinity	Hisat2/StringTie
Mean exon length	371	349	355
Max. exon length	8785	3545	2437
Min. exon length	2	9	13
Median exon length	262	298	303
Mean transcript length	2326	381	524
Mean intron length	2956	470	406
Max. intron length	121,413	3653	3652
Min. intron length	67	65	61
Median intron length	522	156	190

**Table 4:** Similarity between novel maize lncRNA sequences and sequences from external databases.

Sequence Similarity Characteristics	Trinity Assembly	Hisat2/StringTie Assembly
Number of homologous sequences	8808 (14%)	1136 (31%)
Number of homologs with maize sequences (non-conserved)	8165 (13%)	1081 (30%)
Number of homologs with sequences from other species (conserved)	643 (1%)	55 (1%)

## Data Availability

Supplementary data to this article can be found online at https://data.mendeley.com/datasets/fnk8pmp2yz/3 (accessed on 25 April 2023). The source code of the pipeline is freely available on the GitHub website https://github.com/artempronozin95/ICAnnoLncRNA-identification-classification-and-annotation-of-LncRNA (accessed on 25 April 2023).

## Supplementary Materials

The following supporting information can be downloaded at: https://www.mdpi.com/article/10.3390/genes14071331/s1, Table S1: The ICAnnoLncRNA input data description. Table S2: List of the 15 maize transcriptome libraries used for the demonstration of the pipeline analysis. Table S3: The number of sequences for different plant species in the lncRNA compilation from external databases used in the current work. Table S4: Distribution of the number of lncRNAs between different types of clusters obtained by sequence similarity search within external databases at 50% identity threshold. Table S5: The number and the fraction (in parentheses) of the novel maize lncRNAs with similarity to sequences from different species represented in the external databases. Figure S1: Tissue description for the set of the 15 transcriptomic experiments for maize. Figure S2: The structure of the directed acyclic graph (DAG) for the ICAnnoLncRNA pipeline. References [74,75,76] are cited in the Supplementary Materials.

## Abbreviations

ATHA	*Arabidopsis thaliana*
BDIS	*Brachypodium distachyon*
FLC	FLOWERING LOCUS C
FPKM	fragments per kilobase of exon per million mapped fragments
lncRNA	long non-coding RNA
mRNA	messenger RNA
ORF	open reading frame
OSAT	*Oryza sativa*
SBIC	*Sorghum bicolor*
SRA	Sequence Read Archive
TPM	transcripts per million
ZMAY	*Zea mays*
