# Supplementary material for: ICAnnoLncRNA: A Snakemake Pipeline for a Long Non-Coding-RNA Search and Annotation in Transcriptomic Sequences
Source: Genes (Basel). 2023 Jun 24;14(7):1331. doi: 10.3390/genes14071331 (PMC10379598; doi:10.3390/genes14071331)
Supplement: Supplementary file 1 [file genes-14-01331-s001.zip › genes-2398882-supplementary.pdf]

Supplementary file for Pronozin A.Yu. & Afonnikov D.A. “ICAnnoLncRNA: a Snakemake pipeline for a Long-Non-coding-RNA Search and Annotation in Transcriptomic Data”

Table S1. The ICAnnoLncRNA input data description.

Input data	File format	Comments
Transcriptome assembly	FASTA	Transcriptome sequences provided by user. They should be obtained before applying the ICAnnoLncRNA pipeline.
Known mRNA for the species under study	FASTA	We recommend to use sequences from genomic databases, like ENSEMBL, TAIR etc.
Known lncRNA for the species under study	FASTA	
Reference genome	FASTA	
Annotation of the reference genome	GFF	We recommend to use sequences from genomic databases, like ENSEMBL, TAIR etc.
TEs coordinates in the reference genome	BED	This annotation should be obtained by user before applying the ICAnnoLncRNA pipeline. The check for the overlap of predicted lncRNAs with TE loci will be turned off if this file is absent.
Annotation of the transcriptome libraries by tissue type	TSV	See file example in Figure S1
Expression data	TSV	1 column – transcript name, 2 column – expression value (for example, TPM), 3 column – library of that transcript

Table S2. List of 15 maize transcriptome libraries used for the demonstration of the pipeline analysis.

Experiment Accession	Tissue	Experiment description	Reference
SRX339787	ear	6-8 mm from tip of ear primordium 2	[74]
SRX711024	ear	parent inbred lines for three tissue types (ear, leaf and stem)	[75]
SRX710962	ear	parent inbred lines for three tissue types (ear, leaf and stem)	[75]
SRX339763	endosperm	endosperm_crown_r2	[74]
SRX339764	endosperm	endosperm_crown_r2	[74]
SRX339758	endosperm	endosperm_crown_r2	[74]
SRX339796	root	Root elongation zone	[74]
SRX339801	root	Root elongation zone	[74]
SRX339794	root	Root elongation zone	[74]
SRX339808	pollen	Mature pollen 2	[74]
SRX339809	pollen	Mature pollen 2	[74]
SRX339807	pollen	Mature pollen 2	[74]
SRX151746	tassel	e1 tassels	[76]
SRX151748	tassel	e1 tassels	[76]
SRX151751	tassel	e1 tassels	[76]

Table S3. The number of sequences for different plant species in the lncRNA compilation from external databases used in the current work.

Species	PNRD	CANTATAdb	GREENC	PlncDB	EVLncRNA	Total
Medicago truncatula	2	3590	9676	13105	4	26250
Glycine max	-	3096	6689	12576	1	22319
Populus trichocarpa	-	-	-	10556	19	10535
Arabidopsis thaliana	2597	4373	3008	5568	144	12051
Vitis vinifera	2124	10761	18110	12253	26	23236
Oryza sativa japonica	790	2788	5237	5216	40	13880
Brachypodium distachyon	-	-	-	12821	-	12804
Sorghum bicolor	-	2600	5305	27623	-	35400
Zea mays	1	4542	2526	16208	1	39456
Selaginella moellendorffii	-	2267	906	5615	-	8727
Physcomitrella patens	-	1498	9690	19319	-	30404
Ostreococcus tauri	-	-	501	-	-	490
Volvox	-	-	1134	-	-	1128
Amborella trichopoda	-	5511	5698	8215	-	19411

Table S4. Distribution of the number of lncRNAs between different types of clusters obtained by sequence similarity search within external databases at 50% identity threshold.

**Clustering characteristics**	**Trinity assembly**		**Hisat2/StringTie assembly**	
	Number	Sequences per cluster, median	Number	Sequences per cluster, median
Clusters with external sequences	289829	1	244295	1
Clusters with novel sequences only	7439	2	284	2
Clusters with external sequences only	281419	1	243750	1
Clusters with novel and external sequences	971	4	261	4

Table S5. The number and the fraction (in parentheses) of the novel maize lncRNAs with similarity to sequences from different species represented in the external databases.

**Species**	**Trinity assembly**	**Hisat2/StringTie assembly**
Zea mays	8165 (92%)	1026 (94%)
Sorghum bicolor	355 (4%)	31 (2%)
Oryza sativa japonica	109 (1%)	8 (0.7%)
Brachipodium distachion	9 (0.1%)	1 (0.09%)
Arabidopsis thaliana	13 (0.1%)	4 (0.3%)
Vitis vivifera	0	1 (0.09%)
Glycine max	1 (0.004%)	0
Medicago truncatula	1 (0.004%)	0
Populis trichocarpa	0	0
Physcomitrella patens	0	0
Selaginella moellendorfii	0	0
Amborell trichopoda	3 (0.004%)	0
Volvox	1 (0.004%)	1 (0.09%)

ZM	SRX339787	ear
ZM	SRX711024	ear
ZM	SRX339763	endosperm
ZM	SRX710962	ear
ZM	SRX339808	Mature pollen
ZM	SRX339794	root
ZM	SRX151746	tassel primordia
ZM	SRX339764	endosperm
ZM	SRX339801	root
ZM	SRX339758	endosperm
ZM	SRX151748	tassel primordia
ZM	SRX151751	tassel primordia
ZM	SRX339809	Mature pollen
ZM	SRX339807	Mature pollen
ZM	SRX339796	root
ZM	SRX710962	ear
ZM	SRX711024	ear
ZM	SRX151746	tassel primordia
ZM	SRX151748	tassel primordia
ZM	SRX151751	tassel primordia
ZM	SRX339758	endosperm
ZM	SRX339763	endosperm
ZM	SRX339764	endosperm
ZM	SRX339787	ear
ZM	SRX339794	root
ZM	SRX339796	root
ZM	SRX339801	root
ZM	SRX339807	Mature pollen
ZM	SRX339808	Mature pollen
ZM	SRX339809	Mature pollen

Figure S1. Tissue description for the set of the 15 transcriptomic experiments for maize. The file at the the ICAnnoLncRNA input should be in tab separated values format (TSV) and contain species abbreviation in the 1st column, library name in the 2nd column and tissue name in the 3rd column.

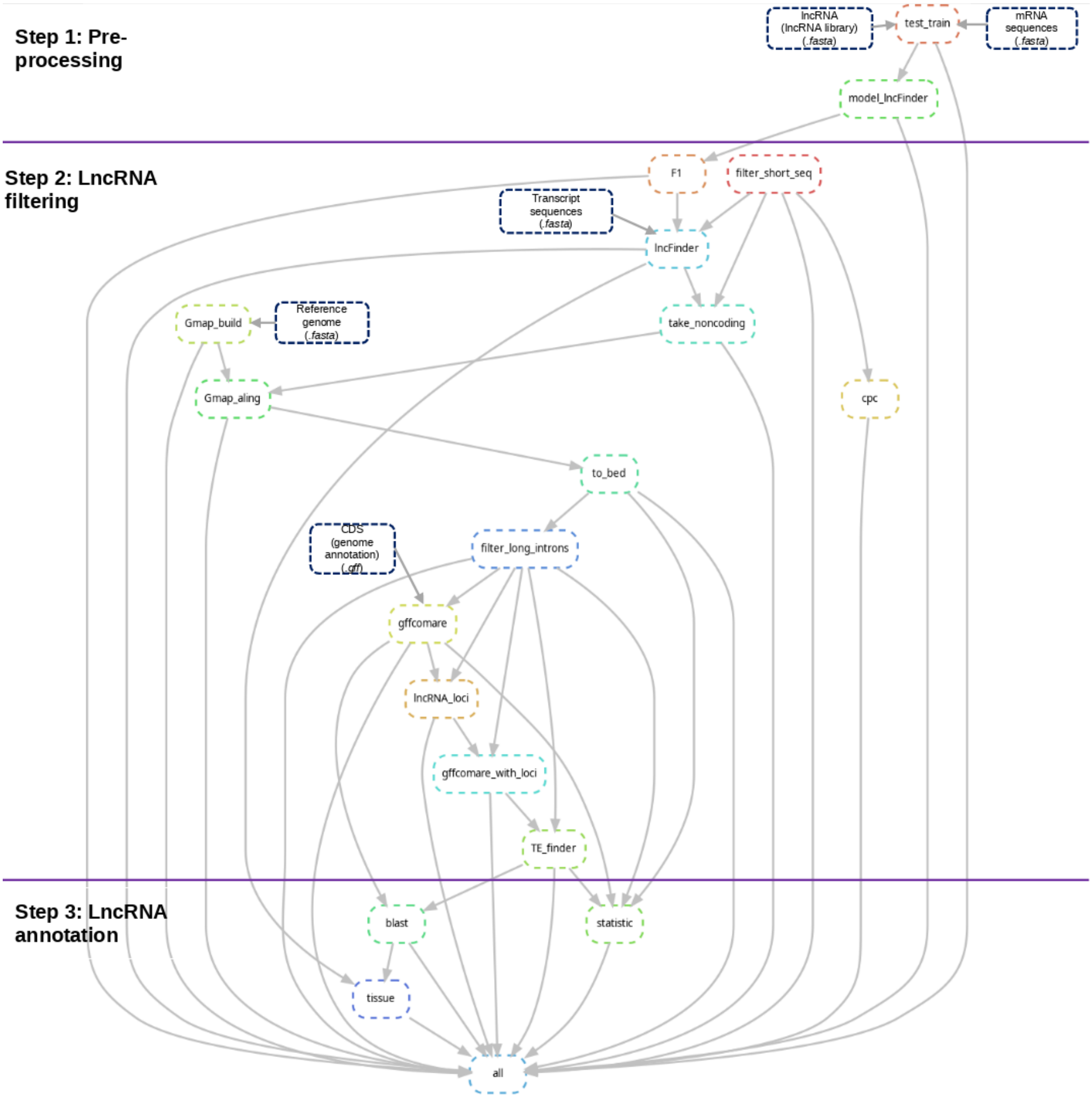

Figure S2. The structure of the directed acyclic graph (DAG) for the ICAnn LncRNA pipeline. Horizontal lines separate three main steps of the analysis.
